# A Combined Injectable and Fractional 1470 nm Laser Approach for the Management of Facial Atrophic Acne Scars: Prospective Ultrasound-Based Evaluation

**DOI:** 10.3390/biomedicines14071441

**Published:** 2026-06-25

**Authors:** Paweł Kubik, Wojciech Gruszczyński, Aleksandra Pawłowska, Maciej Malinowski, Brygida Baran, Agnieszka Pawłowska-Kubik, Łukasz Kodłubański, Bartłomiej Łukasik

**Affiliations:** 1 K-LAB Badania i Rozwój, 81-312 Gdynia, Poland; wojciech.gruszczynski@k-lab.com.pl (W.G.); aleksandra.pawlowska@k-lab.com.pl (A.P.); maciejmal@gmail.com (M.M.); agnieszkapawl@wp.pl (A.P.-K.); 2 Medical Department, Matex Lab Switzerland SA, 1228 Geneva, Switzerland; brygida.baran@neauvia.com (B.B.); bartlomiej.lukasik@neauvia.com (B.Ł.); 3 Department of Human Rights and Intellectual Property Law, University of Gdańsk, 80-309 Gdańsk, Poland; lukasz.kod@gmail.com

**Keywords:** acne scarring, acne scar management, PEGDE-crosslinked HA filler, CaHA, 1470 nm laser

## Abstract

**Background:** Acne vulgaris affects up to 80% of individuals aged 11–30 years and frequently results in permanent scarring with significant psychosocial impact. This prospective single-arm case series evaluated the safety and high-frequency ultrasound-assessed morphological changes in a combined protocol integrating subcision, PEGDE-crosslinked hyaluronic acid supplemented with calcium hydroxyapatite (CaHA), and fractional 1470 nm diode laser therapy in patients with facial atrophic acne scars. **Methods:** Twenty patients (aged 18–42 years, Fitzpatrick phototypes I–II) with moderate-to-severe atrophic acne scars underwent subcision of fibrotic adhesions using a 22G cannula combined with a single subcutaneous injection of 2 mL PEGDE-crosslinked hyaluronic acid with CaHA microparticles on day 0, followed by two sessions of fractional 1470 nm diode laser therapy on days 7 and 28. Scar depth and diameter were assessed using high-frequency ultrasound (48 MHz) at baseline and on days 28, 49, 77, and 139. **Results:** All participants completed the protocol without serious adverse events. High-frequency ultrasound demonstrated progressive reductions in mean scar depth (from 0.35 to 0.05 mm; −86%) and scar diameter (from 4.27 to 1.06 mm; −75%) by day 139, with reductions continuing beyond the active treatment phase. In linear mixed-effects models accounting for within-patient clustering of the two lesions assessed per participant, the reductions in both depth and diameter were statistically significant at every follow-up timepoint relative to baseline (all *p* < 0.001). These ultrasound findings were not corroborated by a control group, blinded assessment, validated clinical grading, or patient-reported outcomes. **Conclusions:** In this single-arm case series, the combined subcision, PEGDE-crosslinked HA–CaHA filler, and fractional 1470 nm diode laser protocol was well tolerated and associated with progressive, sustained reductions in high-frequency ultrasound-measured scar depth and diameter. As an uncontrolled, unblinded study without validated clinical grading or patient-reported outcomes, these findings are preliminary and require confirmation in larger, controlled trials.

## 1. Introduction

Acne vulgaris remains one of the most prevalent inflammatory skin disorders worldwide, affecting up to 80% of individuals between the ages of 11 and 30 [1,2]. While peak incidence occurs during mid-adolescence, typically earlier in females than in males [2], an increasing prevalence has been observed among prepubescent patients in recent years [3], alongside persistence of the condition well into adulthood in a clinically significant subset of individuals [1,4]. Although many cases resolve within several years, a proportion of patients experience prolonged disease activity extending beyond a decade [5]. The pathogenesis of acne is driven by a multifactorial process involving sebaceous gland hyperactivity, follicular hyperkeratinisation, *Cutibacterium acnes* proliferation, and subsequent inflammatory cascades [1,2]. When inadequately controlled, acne-related inflammation may extend into the dermis, resulting in post-inflammatory dyspigmentation and permanent scarring. Atrophic scars are predominantly observed on the face and upper back, whereas hypertrophic scars and keloids more frequently involve the chest and shoulders [1,6]. Facial scarring has been reported in up to 95% of affected individuals, although this figure encompasses all grades of severity, including subclinical forms [7]. Importantly, delayed initiation of appropriate therapy is consistently associated with increased scar severity [5]. Unlike active acne lesions, residual scars do not undergo spontaneous resolution and therefore represent a long-term structural alteration of the dermal matrix [7,8].

Beyond their physical manifestation, acne scars are associated with substantial psychosocial burden, contributing to reduced self-confidence, social withdrawal, and anxiety [9,10]. A multinational, mixed-methods study by Tan et al. [11] demonstrated that patients with facial acne scars reported significant impairment across emotional, social, and functional domains, with 17% displaying overconcern with scar appearance and adopting avoidance behaviours. Among those with severe scarring, approximately 69% reported meaningful impairment in dermatology-related quality of life [12]. These findings have been further amplified in the context of appearance-focused digital environments [13]. These data underscore that the burden of acne scarring extends well beyond cosmetic concern and constitutes a clinically relevant indication for effective, timely intervention.

Clinically, atrophic acne scars are classified into rolling, boxcar, and icepick subtypes, each reflecting distinct patterns of dermal collagen loss [6,14]. Rolling scars present as undulating depressions tethered by fibrous septae, boxcar scars exhibit sharply demarcated vertical edges, and icepick scars extend deeply and narrowly into the dermis. Because each subtype reflects a distinct depth and architecture of tissue damage, effective management requires subtype-specific intervention strategies that may combine subcision, dermal fillers, and resurfacing techniques within a multimodal approach [6,14,15]. Among energy-based therapies, laser-based modalities have been reported to improve scar texture and depth, although deeply penetrating icepick scars remain particularly resistant to treatment [14]. Ablative fractional lasers, in particular fractional CO_2_ (10,600 nm) and Er:YAG (2940 nm), are considered first-line energy-based modalities for moderate-to-severe atrophic scars [16]. However, they are associated with a clinically relevant risk of post-inflammatory hyperpigmentation, prolonged social downtime, and procedural discomfort, which limits their suitability in patients with higher Fitzpatrick phototypes or restricted recovery time [17,18]. Non-ablative fractional 1470 nm diode lasers offer an alternative approach by creating microthermal columns within the dermis while preserving epidermal integrity, thereby enabling controlled collagen remodelling with a markedly more favourable safety profile, albeit typically requiring a greater number of treatment sessions to achieve comparable outcomes [19,20].

Dermal fillers directly address the volumetric deficit underlying atrophic scars. PEGDE-crosslinked hyaluronic acid formulations provide immediate structural support with favourable rheological and biocompatibility properties [21,22]. The addition of calcium hydroxyapatite (CaHA) microparticles confers an important biostimulatory dimension: CaHA activates dermal fibroblasts and induces neocollagenesis, significantly increasing the proportion of newly formed type III collagen in treated tissue, contributing to structural reinforcement that extends beyond immediate volumisation and persists over several months [23,24]. This dual action, combining volumetric correction with progressive extracellular matrix remodelling, makes CaHA-enriched fillers a mechanistically well-suited adjunct to both mechanical and laser-based interventions.

Although subcision, dermal fillers, and fractional laser therapy have each been studied individually or in pairwise combinations [25,26], no prospective study to date has evaluated the simultaneous integration of all three modalities, namely mechanical fibrotic release, biostimulatory injectable scaffold, and controlled non-ablative thermal remodelling, within a single stepwise protocol, particularly with respect to objective instrumental outcomes such as high-frequency ultrasound (HFUS) assessment [27,28]. The purpose of this prospective, single-arm case series was therefore to explore the safety and HFUS-assessed morphological changes associated with a combined protocol using subcision, PEGDE-crosslinked hyaluronic acid supplemented with calcium hydroxyapatite, and fractional 1470 nm diode laser therapy in patients with facial atrophic acne scars. Given its uncontrolled and exploratory design, the study was intended to generate preliminary data to inform subsequent controlled trials rather than to establish clinical efficacy.

## 2. Materials and Methods

### 2.1. Study Design and Participants

This was a prospective, single-arm, interventional (prospective case series) study. The study was approved by the Ethics Committee of the Medical Chamber in Gdańsk, Poland (KB-(10)17/2023/24.01.2023) and conducted in accordance with the principles of the Declaration of Helsinki. All participants provided written informed consent prior to enrolment. Twenty patients aged 18–42 years presenting with moderate-to-severe atrophic facial acne scars were enrolled. Scar severity was categorised as moderate-to-severe by the treating physician using the qualitative Goodman & Baron global acne-scarring grading scale. Inclusion criteria were Fitzpatrick skin phototype I–II and the presence of atrophic facial acne scars of any subtype (rolling, boxcar, or icepick). Exclusion criteria included active inflammatory acne, recent systemic retinoid therapy (within the preceding 6 months), excessive ultraviolet exposure within 4 weeks prior to enrolment, history of keloidal scarring, pregnancy or lactation, and clinically significant medical comorbidities.

### 2.2. Materials

#### 2.2.1. Injectable Filler

Neauvia Stimulate (Matex Lab, Geneva, Switzerland) is a monophasic, PEGDE-crosslinked hyaluronic acid hydrogel containing stabilised sodium hyaluronate (26 mg/mL) combined with 1% calcium hydroxyapatite (CaHA) microparticles (10–12 µm diameter) and enriched with glycine and L-proline in a pyrogen-free buffered solution. Crosslinking with polyethylene glycol diglycidyl ether (PEGDE) provides favourable rheological properties and high biocompatibility [21,22]. In addition to immediate volumisation, the CaHA microparticles are intended to support progressive dermal remodelling through fibroblast activation and neocollagenesis [24].

#### 2.2.2. Laser Device

The LaserMe 1470 nm diode system (Berger & Kraft Medical Sp. z o.o., Warsaw, Poland) is a fractional non-ablative device emitting monochromatic radiation at 1470 nm with a maximum power output of 2 W. The system delivers energy in multi-millisecond pulses with an adjustable energy range of 5–50 mJ per microbeam. The fractional non-ablative mechanism generates controlled microthermal columns within the dermis while preserving epidermal integrity, enabling collagen remodelling with minimal downtime [19,20].

### 2.3. Treatment Protocol

The treatment protocol comprised three sequential components delivered over 28 days.

Day 0—Subcision and filler injection.

Fibrotic adhesions were released using a 22G blunt cannula introduced through a limited number of entry points at the deep dermal to superficial subcutaneous level. Controlled fanning movements were used to mechanically detach tethered scar tissue from the underlying plane. In the same session, 2 mL of Neauvia Stimulate was injected into the subcutaneous plane beneath the treated scars to provide immediate structural support and serve as a biostimulatory scaffold for subsequent tissue remodelling.

Day 7—First laser session.

Fractional 1470 nm diode laser therapy was administered to the treated areas. Settings were individualised for each patient according to scar severity, depth, and skin response, and were adjusted at each session according to patient tolerance; parameters could therefore differ between the two laser sessions. Across all treated areas, 2–3 passes were performed per session, with energy per microbeam (MTZ) of 24–35 mJ and microbeam spacing of 1.4–2.0 mm. The clinical treatment endpoint was moderate erythema of the treated skin during the procedure.

Day 28—Second laser session.

The day 28 follow-up ultrasound assessment was performed before the second laser session. A second session of fractional 1470 nm diode laser therapy was then delivered using the same clinical endpoint and individualised adjustment strategy.

### 2.4. Follow-Up and Outcome Assessment

Clinical and instrumental assessments were performed at baseline (day 0) and during four follow-up visits on days 28, 49, 77, and 139 after the initial treatment.

#### 2.4.1. Primary Outcome Measure

**Figure 1:**
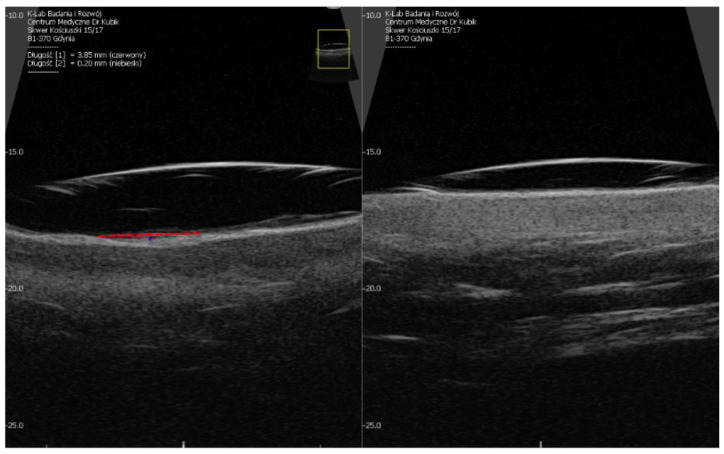
Representative high-frequency ultrasound (HFUS, 48 MHz) images of an atrophic acne scar before (**left**) and after (**right**) the combined treatment protocol. Prior to treatment, the scar depression is clearly delineated, with a transverse diameter of 3.85 mm (red marker) and a depth of 0.20 mm (blue marker). The ultrasound image was acquired with the depth scale displayed in millimetres, ranging approximately from 10 to 25 mm. At day 139, the scar contour is no longer discernible, consistent with structural normalisation of the dermal profile. A subtle difference in dermal echogenicity at the previously affected site reflects ongoing tissue remodelling.

Scar depth and transverse scar diameter (width) were measured using a high-frequency ultrasound system operating at 48 MHz (Dramiński Technology, Sząbruk, Poland). For each participant, two representative atrophic scars were selected at baseline and consistently evaluated throughout the follow-up period by the same investigator. The two scars assessed per participant were the atrophic scars of the largest diameter that were simultaneously accessible to reliable high-frequency ultrasound examination (Figure 1). When more than two scars met these criteria, the two lesions with the largest diameter and reliable acoustic access were selected to maintain a consistent two-lesion-per-participant assessment strategy. Scars of all three atrophic subtypes (rolling, boxcar, and icepick) were eligible for inclusion; lesions were analysed in aggregate and were not stratified by subtype. Scar depth was defined as the vertical distance from the epidermal surface of adjacent healthy skin to the deepest point of the scar depression. Scar diameter was defined as the horizontal distance between the outermost edges of healthy epidermis across the scar. Measurement precision was ±0.01 mm. For descriptive tables and figures, percentage change from baseline was calculated from unrounded group means as [(follow-up mean − baseline mean)/baseline mean] × 100.

#### 2.4.2. Safety Assessment

Adverse events were monitored at each follow-up visit and classified by type, severity, and duration. Participants were additionally instructed to report any unexpected symptoms between scheduled visits.

### 2.5. Statistical Analysis

Each of the 20 participants contributed two scars, and each scar was assessed at five timepoints, yielding 40 lesions and 200 observations per ultrasound outcome. Continuous outcomes (scar depth and scar diameter) are summarised as mean, standard deviation (SD), and 95% confidence interval (CI); median and interquartile range (IQR) were additionally examined. Changes over time were analysed using linear mixed-effects models with timepoint as a categorical fixed effect (baseline as the reference) and random intercepts for participant and for scar nested within participant, thereby accounting for repeated measurements over time and within-patient clustering of the two lesions assessed per participant. The overall effect of time was assessed using a likelihood-ratio test comparing maximum-likelihood (ML) models with and without time as a fixed effect. The final model was then refitted using restricted maximum likelihood (REML), and each follow-up timepoint was compared with baseline using model-estimated mean differences with 95% CIs and *p*-values. A two-sided *p*-value < 0.05 was considered statistically significant. Analyses were performed in Python 3 using the statsmodels package (version 0.14).

## 3. Results

### 3.1. Clinical and Safety Outcomes

All 20 enrolled patients completed the study protocol without interruption. No serious or persistent adverse events were recorded. Transient peri-procedural erythema was anticipated and constituted the intended clinical treatment endpoint. Clinically, patients demonstrated progressive improvement in overall skin texture and softening of atrophic scar contours during subsequent follow-up visits, consistent with the objective ultrasound findings described below.

### 3.2. High-Frequency Ultrasound Outcomes

High-frequency ultrasound (HFUS) imaging was employed to assess structural changes in acne scars before and after treatment. For each participant, two representative atrophic scars were selected for serial analysis, yielding a total of 40 evaluated lesions. All evaluations were performed using a 48 MHz ultrasound system (Dramiński Technology, Poland). Scar depth and transverse diameter were quantified in millimetres with a measurement precision of 0.01 mm. Repeated measurements were obtained at each timepoint to ensure consistency.

#### 3.2.1. Scar Depth

**Figure 2:**
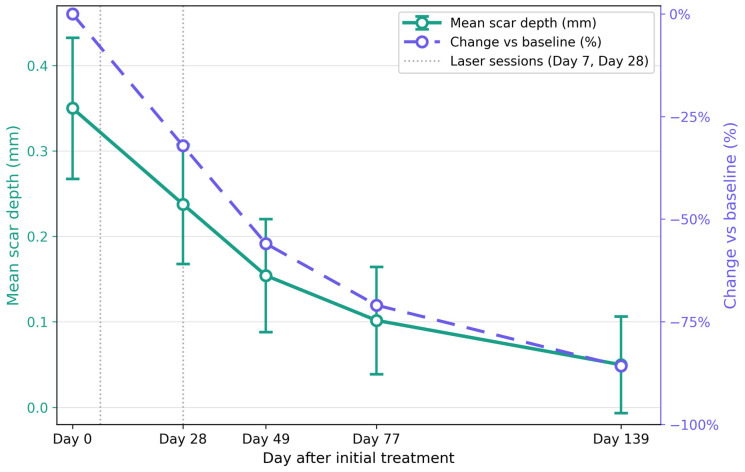
Temporal changes in mean HFUS-measured scar depth and percentage reduction from baseline following combined subcision, PEGDE-crosslinked HA–CaHA filler, and fractional 1470 nm diode laser treatment (40 scars from 20 participants). Solid line (left y-axis): absolute mean scar depth in millimetres. Dashed line (right y-axis): percentage change relative to baseline. Vertical dotted lines indicate fractional 1470 nm diode laser sessions on days 7 and 28. The day 28 ultrasound assessment was performed before the second laser session. Error bars represent sample SD across 40 scars. All *p* < 0.001 vs. baseline.

Mean scar depth at baseline was 0.35 mm. A progressive reduction was observed at each subsequent follow-up visit (Table 1; Figure 2). By day 28, corresponding to 21 days after the first fractional 1470 nm diode laser session, mean depth had decreased to 0.24 mm (−32% from baseline). Further reductions were documented at day 49 (0.15 mm; −56%) and day 77 (0.10 mm; −71%). Notably, improvement continued well beyond the active treatment phase: at day 139, corresponding to 111 days after the final (day 28) laser session, mean scar depth had decreased to 0.05 mm, representing an overall reduction of 86% from baseline. The steepest absolute decline occurred between baseline and day 28 (−0.11 mm), whereas the rate of reduction gradually tapered during the follow-up period, consistent with ongoing but decelerating dermal remodelling. In the linear mixed-effects model accounting for repeated measurements and within-patient clustering, the reduction in scar depth was statistically significant at every follow-up timepoint relative to baseline (overall effect of time, *p* < 0.001), with model-estimated mean differences of −0.11 mm (95% CI −0.13 to −0.10) at day 28, −0.20 mm (95% CI −0.21 to −0.18) at day 49, −0.25 mm (95% CI −0.26 to −0.23) at day 77, and −0.30 mm (95% CI −0.32 to −0.29) at day 139 (all *p* < 0.001).

#### 3.2.2. Scar Diameter (Width)

**Figure 3:**
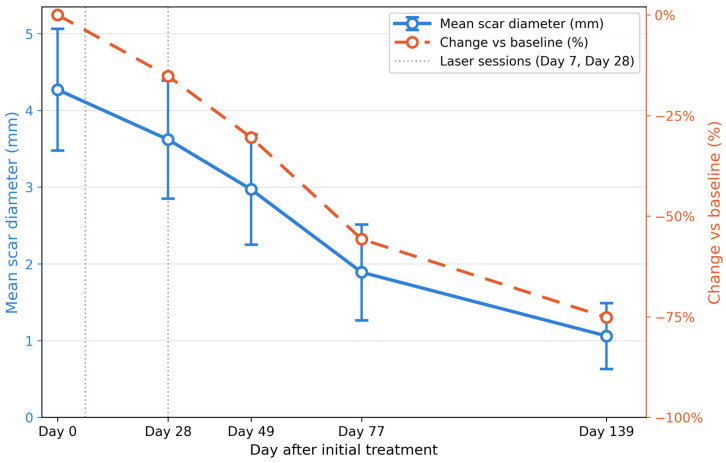
Temporal changes in mean HFUS-measured scar diameter and percentage reduction from baseline following combined subcision, PEGDE-crosslinked HA–CaHA filler, and fractional 1470 nm diode laser treatment (40 scars from 20 participants). Solid line (left y-axis): absolute mean scar diameter in millimetres. Dashed line (right y-axis): percentage change relative to baseline. Vertical dotted lines indicate fractional 1470 nm diode laser sessions on days 7 and 28. The day 28 ultrasound assessment was performed before the second laser session. Error bars represent sample SD across 40 scars. All *p* < 0.001 vs. baseline.

**Figure 4:**
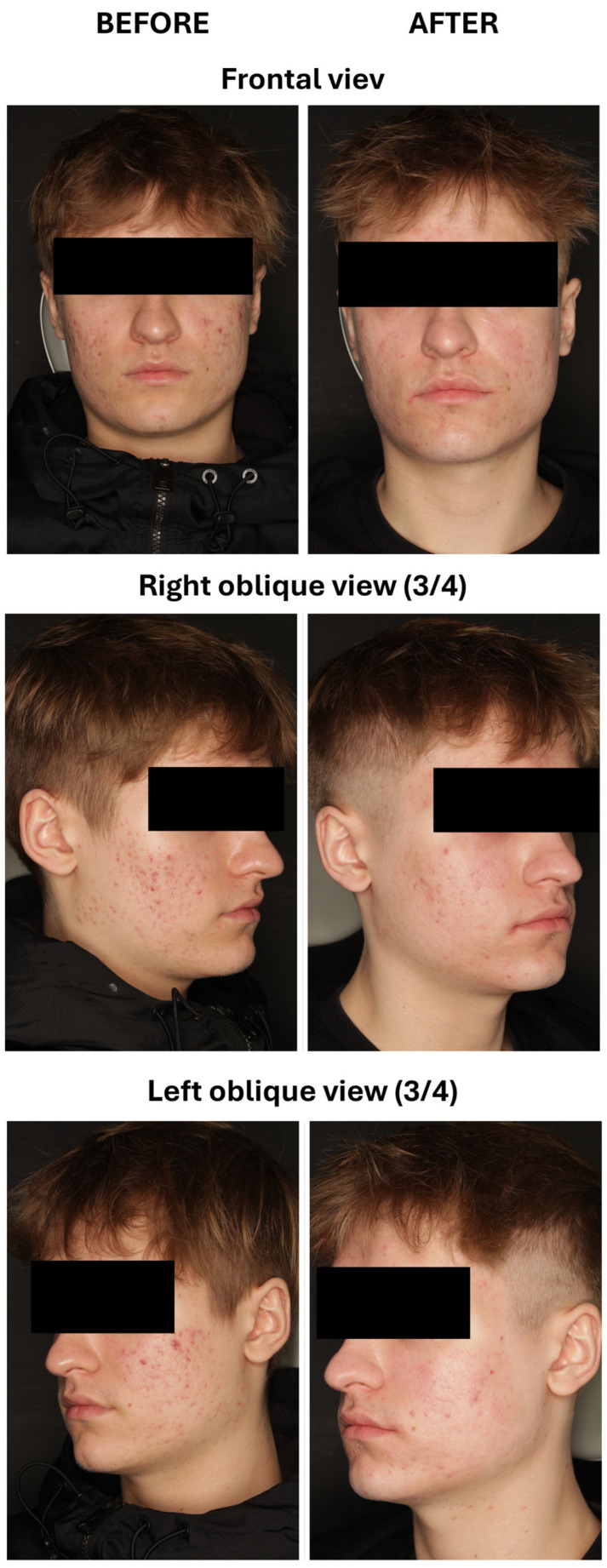
Representative clinical photographs of a patient with facial atrophic acne scars before (BEFORE) and after (AFTER) completion of the combined treatment protocol, shown in three standardised projections: frontal, right oblique (¾), and left oblique (¾). Eyes obscured to protect patient privacy. Marked overall improvement in skin texture and reduction in atrophic scar contours is visible across all projections. Written informed consent for publication of clinical images was obtained from the patient.

Mean scar diameter at baseline was 4.27 mm. Similar to scar depth, a progressive reduction was observed across all follow-up visits (Table 2; Figure 3). At day 28, mean diameter decreased to 3.62 mm (−15%), with continued reductions at day 49 (2.97 mm; −30%) and day 77 (1.89 mm; −56%). At the final assessment on day 139, the mean diameter was 1.06 mm, corresponding to a 75% reduction from baseline. In the linear mixed-effects model accounting for repeated measurements and within-patient clustering, the reduction in scar diameter was statistically significant at every follow-up timepoint relative to baseline (overall effect of time, *p* < 0.001), with model-estimated mean differences of −0.65 mm (95% CI −0.74 to −0.56) at day 28, −1.30 mm (95% CI −1.39 to −1.21) at day 49, −2.38 mm (95% CI −2.47 to −2.29) at day 77, and −3.21 mm (95% CI −3.30 to −3.12) at day 139 (all *p* < 0.001). Interestingly, the temporal pattern of diameter reduction differed from that of scar depth. Whereas the most pronounced absolute decrease in depth occurred during the early treatment phase (baseline to day 28), the steepest decline in scar diameter was observed between day 49 and day 77 (−1.08 mm; −26 percentage points). Representative standardised clinical photographs illustrating the overall improvement in scar appearance across three facial projections are presented in Figure 4.

## 4. Discussion

The present prospective single-arm case series evaluated longitudinal changes in high-frequency ultrasound (HFUS)-measured scar depth and diameter following a combined protocol of subcision, PEGDE-crosslinked hyaluronic acid supplemented with calcium hydroxyapatite (CaHA) microparticles, and fractional non-ablative 1470 nm diode laser therapy for atrophic facial acne scars. Progressive reductions in both ultrasound-measured outcomes were observed through day 139. No serious or persistent adverse events were recorded in this cohort. However, because all participants received the same multi-component intervention and no comparator group was included, the findings should be interpreted as descriptive of the treated cohort and do not establish clinical efficacy or causality.

The temporal patterns of the two ultrasound outcomes differed. The largest absolute reduction in scar depth occurred between baseline and day 28, whereas the largest reduction in scar diameter occurred between days 49 and 77. The day 28 assessment was performed after subcision and filler injection on day 0 and after the first laser session on day 7, but before the second laser session. The later reduction in scar diameter occurred after completion of the active treatment phase. These temporal differences may be compatible with different phases of tissue response, including early mechanical release and volume support followed by later remodelling processes [29,30]. However, they do not establish the relative contribution of subcision, filler injection, or laser treatment to the observed changes.

The delayed reduction in scar diameter may be compatible with a progressive remodelling response. Previous histological and clinical studies suggest that CaHA microparticles may stimulate fibroblast activity and collagen synthesis over several weeks [23,24]. Cavallini et al. [31] reported progressive dermal thickening on HFUS after injection of PEGDE-crosslinked HA with CaHA, with changes observed at 30 days, 4 months, and 9 months. These reports provide a biological rationale for considering a delayed tissue response after CaHA-containing filler injection. However, collagen synthesis, tissue contraction, persistence of filler material, and component-specific effects were not measured in the present study. Therefore, a CaHA-mediated biostimulatory mechanism remains hypothesis-generating rather than demonstrated.

The combined protocol was designed to address several structural features of atrophic acne scars through complementary approaches. Subcision is intended to release fibrous septae that tether the scar base to underlying tissue and may initiate a local wound-healing response. A systematic review by Ahramiyanpour et al. [32] reported that both needle-based and cannula-based subcision may be effective for atrophic acne scars, with blunt cannula techniques potentially associated with fewer adverse effects and greater patient satisfaction in selected settings. A comprehensive review by Vempati et al. [33] also highlighted the potential value of combining subcision with injectable materials, including hyaluronic acid gels, to support the released tissue and reduce recurrent depression of the scar base. Nevertheless, the present study did not include a subcision-only group and cannot determine the independent contribution of subcision to the observed ultrasound changes.

The injectable component may provide immediate volume support beneath selected scars. In a prospective placebo-controlled trial, Siperstein et al. [34] reported improvement in rolling acne scars after treatment with hyaluronic acid filler, with results maintained during follow-up. PEGDE-crosslinked HA may also act as a temporary scaffold within the treated tissue and may influence the local mechanical environment during extracellular matrix remodelling [29,30]. The addition of CaHA microparticles provides a biological rationale for a possible longer-term tissue response [23,24]. Cavallini et al. [31] proposed a bio-regenerative framework in which PEGDE-crosslinked HA combined with low-concentration CaHA may provide immediate volumisation together with delayed biological effects. However, the present study did not include an HA-only group, a CaHA-free filler group, or a no-filler control. It is therefore not possible to determine whether the observed HFUS changes reflected mechanical release, filler-related volume restoration, tissue remodelling, laser-related effects, or a combination of these factors.

The 1470 nm wavelength was selected because water absorption at this wavelength permits controlled dermal heating while preserving the epidermis. Fractional non-ablative 1470 nm laser therapy creates microthermal treatment zones that may promote collagen remodelling [19,20]. Previous studies have reported gradual improvement in acne scar appearance after treatment with 1470 nm laser systems, often after multiple sessions [19,20]. In the present study, the laser was administered after subcision and filler injection as part of the planned treatment sequence. However, the study design does not permit assessment of whether the laser added benefit beyond the injectable and subcision components or whether the timing of the laser sessions influenced the observed outcomes.

The numerical magnitude of the observed reductions in HFUS-measured scar depth and diameter was large. However, these changes should not be interpreted as validated clinical effect sizes because the study did not include blinded clinical assessment, a validated longitudinal scar-grading scale, or patient-reported outcome measures. Direct comparison with published studies is limited by differences in scar subtype, participant characteristics, outcome measures, laser modality, treatment protocol, and follow-up duration.

Tran et al. [25] evaluated subcision combined with fractional CO_2_ laser therapy in a split-face study and reported significant improvement using ECCA scoring and HFUS-measured dermal thickness. Abdelwahab et al. [26] compared subcision combined with fractional CO_2_ laser therapy or cross-linked HA filler with subcision alone and found that both combinations performed better than subcision alone. These studies support further investigation of multimodal treatment strategies. However, they do not permit conclusions regarding the relative contribution or comparative effectiveness of the present three-component protocol, which used a non-ablative 1470 nm laser and a PEGDE-crosslinked HA filler containing CaHA microparticles. Prospective evidence for this specific combination remains limited.

A recent systematic review and network meta-analysis by Wu et al. [15] found that combinations of laser treatment and filler injection ranked highly for reducing clinical acne scar severity scores. This evidence provides a rationale for investigating combined approaches. However, direct comparison with the present findings is not possible because the included studies used heterogeneous treatment protocols, predominantly involved other laser modalities, and evaluated clinical grading outcomes rather than HFUS-measured scar depth and diameter.

A recent Italian expert consensus provided practical recommendations regarding the selection and use of PEGDE-crosslinked HA hydrogels in facial injectable procedures [35]. Such recommendations may support procedural standardisation, but do not provide evidence for the effectiveness of the present combined protocol in atrophic acne scars. Similarly, preliminary reports have described the use of 1470 nm laser treatment in patients with higher Fitzpatrick skin phototypes, including phototype V [36]. However, the present cohort included only patients with Fitzpatrick skin phototypes I–II and does not permit conclusions regarding pigmentary safety or effectiveness in more diverse populations.

This study has several limitations. First, the single-arm, uncontrolled design does not allow causal attribution of the observed changes to the intervention or to any individual treatment component. Second, only two scars per participant were selected for serial assessment. These were the largest scars with reliable acoustic access at baseline, which may limit the representativeness of the overall facial scar burden and may introduce selection bias. Third, HFUS measurements were performed by the same investigator, without blinded longitudinal outcome assessment. In addition, ultrasound measurements cannot distinguish persistent filler-related volume correction from endogenous tissue remodelling.

Scars were analysed in aggregate rather than stratified by rolling, boxcar, and icepick subtype. Consequently, subtype-specific responses could not be determined. Laser settings were individualised and could differ between sessions, reflecting clinical practice but limiting protocol standardisation and preventing assessment of dose–response relationships. No validated longitudinal clinical grading instrument, such as the ECCA scale [37] or Goodman and Baron grading [38], was used, and no patient-reported outcome measures were collected. Although representative clinical photographs were provided, they were not assessed by blinded evaluators and were not analysed with a validated photographic scale. The modest sample size limited precision and was insufficient to assess uncommon adverse events. The follow-up period of 139 days also does not establish long-term durability. Finally, the cohort was limited to Fitzpatrick skin phototypes I–II, which restricts the generalisability of the findings, particularly with respect to pigmentary safety in higher phototypes.

Future studies should use randomised controlled or split-face designs with appropriate comparator groups. Such studies should include blinded clinical assessment, validated scar-grading instruments, patient-reported outcomes, predefined and standardised laser parameters, subtype-specific analyses, reproducibility assessment for HFUS measurements, longer follow-up, and recruitment of participants across a broader range of Fitzpatrick skin phototypes.

In conclusion, in this prospective single-arm case series, the combined protocol of subcision, PEGDE-crosslinked HA with CaHA microparticles, and fractional 1470 nm diode laser therapy was associated with progressive reductions in HFUS-measured scar depth and diameter through day 139, and no serious or persistent adverse events were observed. These uncontrolled findings do not establish clinical efficacy, component-specific effects, synergy between treatment modalities, or comparative safety. Larger controlled studies are needed to determine the clinical relevance, durability, and relative contribution of each component of this multimodal approach.

## Figures and Tables

**Table 1:** Scar depth measured by high-frequency ultrasound (HFUS). Data are unadjusted lesion-level mean, standard deviation (SD), and 95% confidence interval (CI) across 40 lesions (two per participant; n = 20).

Timepoint	Clinical Context	Mean Scar Depth (mm)	SD (mm)	95% CI (mm)	Change vs. Baseline (%)
Day 0	Baseline (pre-treatment)	0.35	0.08	0.32–0.38	–
Day 28	21 days post 1st laser session *	0.24	0.07	0.22–0.26	−32
Day 49	21 days post 2nd laser session *	0.15	0.07	0.13–0.18	−56
Day 77	49 days post 2nd (final) laser session	0.10	0.06	0.08–0.12	−71
Day 139	111 days post 2nd (final) laser session	0.05	0.06	0.03–0.07	−86

* Laser sessions refer to fractional 1470 nm diode laser therapy, administered on day 7 and day 28 of the treatment protocol. All patients (n = 20) underwent subcision with PEGDE-crosslinked hyaluronic acid filler supplemented with calcium hydroxyapatite (CaHA) microparticles on day 0, followed by two laser sessions. Scar depth was assessed using HFUS. Percentage change was calculated from unrounded group means as [(follow-up mean − baseline mean)/baseline mean] × 100. All *p* < 0.001 vs. baseline.

**Table 2:** Scar diameter (width) measured by high-frequency ultrasound (HFUS). Data are unadjusted lesion-level mean, standard deviation (SD), and 95% confidence interval (CI) across 40 lesions (two per participant; n = 20).

Timepoint	Clinical Context	Mean Scar Diameter (mm)	SD (mm)	95% CI (mm)	Change vs. Baseline (%)
Day 0	Baseline (pre-treatment)	4.27	0.79	4.02–4.52	–
Day 28	21 days post 1st laser session *	3.62	0.77	3.37–3.87	−15
Day 49	21 days post 2nd laser session *	2.97	0.72	2.74–3.20	−30
Day 77	49 days post 2nd (final) laser session	1.89	0.62	1.69–2.09	−56
Day 139	111 days post 2nd (final) laser session	1.06	0.43	0.92–1.20	−75

* Laser sessions refer to fractional 1470 nm diode laser therapy, administered on day 7 and day 28 of the treatment protocol. All patients (n = 20) underwent subcision with PEGDE-crosslinked hyaluronic acid filler supplemented with calcium hydroxyapatite (CaHA) microparticles on day 0, followed by two laser sessions. Scar diameter (width) was assessed using HFUS. Percentage change was calculated from unrounded group means as [(follow-up mean − baseline mean)/baseline mean] × 100. All *p* < 0.001 vs. baseline.

## Data Availability

The data presented in this study are available upon request from the corresponding author. The data are not publicly available due to privacy restrictions.
